# Supplementary material for: Arrayed single-gene perturbations identify drivers of human anterior neural tube closure
Source: bioRxiv. 2025 Jul 22:2025.07.21.665862. Preprint. [Version 1] doi: 10.1101/2025.07.21.665862 (PMC12330715; doi:10.1101/2025.07.21.665862)
Supplement: Supplement 1 [file NIHPP2025.07.21.665862v1-supplement-1.pdf]

bioRxiv preprint doi: https://doi.org/10.1101/2025.07.21.665862; this version posted July 22, 2025. The copyright holder for this preprint (which was not certified by peer review) is the author/funder, who has granted bioRxiv a license to display the preprint in perpetuity. It is made available under aCC-BY 4.0 International license.

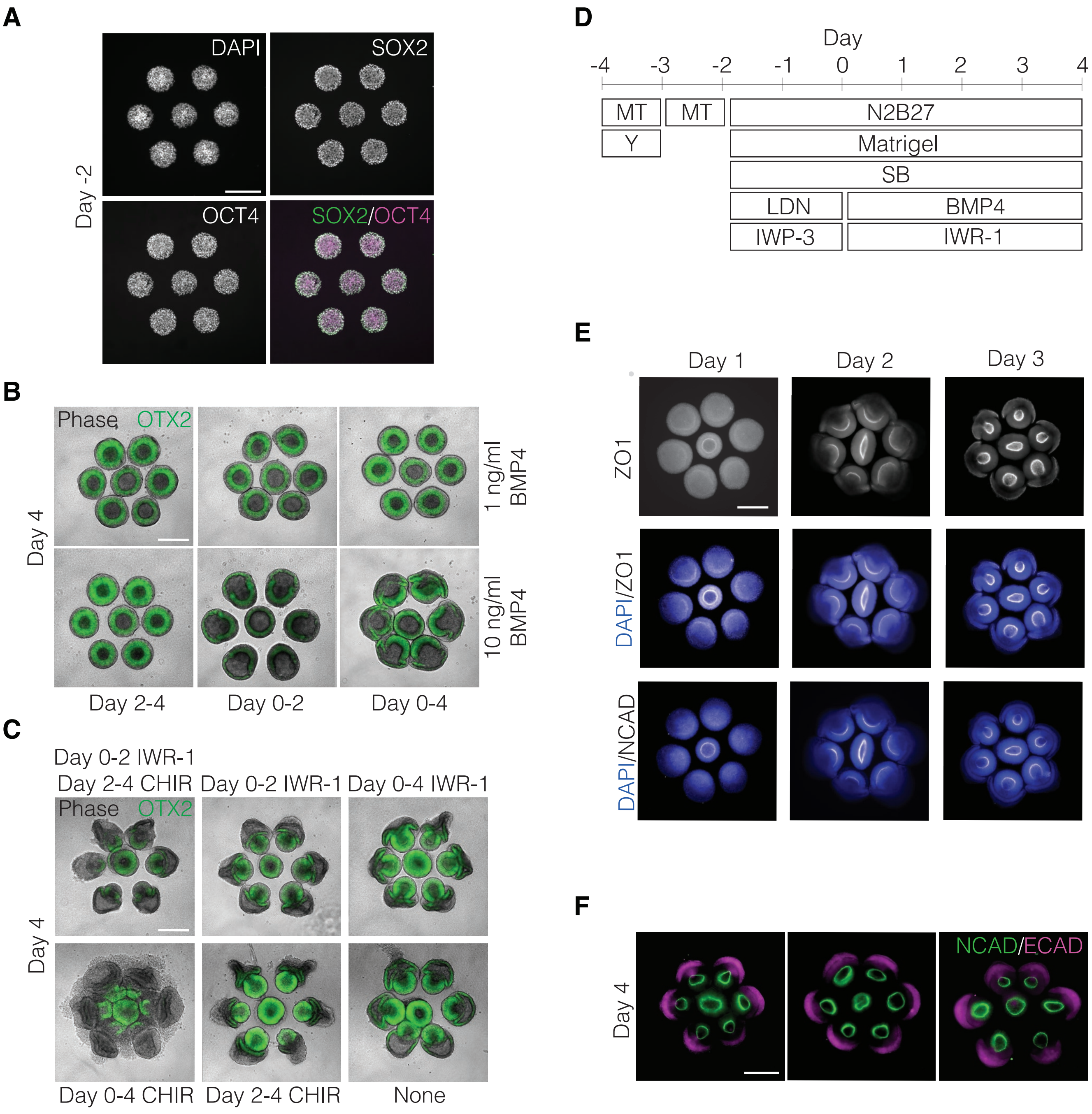

Figure S1

bioRxiv preprint doi: https://doi.org/10.1101/2025.07.21.665862; this version posted July 22, 2025. The copyright holder for this preprint (which was not certified by peer review) is the author/funder, who has granted bioRxiv a license to display the preprint in perpetuity. It is made available under aCC-BY 4.0 International license.

Figure S1. Development and validation of a hPSC-derived *in vitro* model of anterior neurulation.

A) Epifluorescence images of fixed and stained hPSCs 2 days after seeding (Day -2 according to scheme in S1D) onto hexagonally arranged 250-µm diameter micropatterns with 100-µm spacing (Methods), showing co-expression of pluripotency markers SOX2 and OCT4. Scale bar: 300 µm.

B) Live epifluorescence images of neural tube organoids expressing an OTX2-Citrine reporter to evaluate responses to different BMP4 treatment courses. The bottom right condition (Day 0-4 BMP4, 10 ng/ml) was selected for further experiments. Scale bar: 300 µm.

C) Live epifluorescence images of neural tube organoids expressing an OTX2-Citrine reporter to evaluate responses to different WNT activation and inhibition courses with agonist CHIR99021 (CHIR) and inhibitor IWR-1-endo (IWR-1). The top right condition (Day 0-4 IWR-1, 1 µM) was selected for further experiments. Scale bar: 300 µm.

D) Neural tube organoid differentiation protocol. hPSCs are seeded onto 2D micropatterns on Day -4 (2 days prior to differentiation) in mTeSR Plus (MT) with 10 µM ROCK inhibitor Y-27632 (Y). Media is changed to MT without Y on Day -3. On Day -2, organoids are folded into 3D cysts in high-concentration Matrigel and simultaneously differentiated toward ectodermal fates in N2B27 media (Methods) by addition of 10 µM TGFB inhibitor SB431542 (SB), 0.5 µM BMP inhibitor LDN193189 (LDN), and 1 µM WNT inhibitor IWP-3 on Day -2. On Day 0, addition of 10 ng/ml BMP4 induces mediolateral patterning of the ectoderm, while WNT inhibition is sustained by 1 µM IWR-1 addition. BMP4-containing media is replaced on Day 2. Each media replacement includes fresh high-concentration Matrigel.

E) Epifluorescence images of organoids after 1, 2, and 3 days of BMP4 treatment, fixed and stained for neural ectoderm marker NCAD, tight junction marker ZO1, and nuclear marker DAPI. Scale bar: 300 µm.

F) Epifluorescence images of three biological replicates of neural tube organoids on Day 4, showing complete closure in the form of a continuous ring of NCAD. 100% of organoids show closure (see Figure 1D). Scale bar: 300 µm.

bioRxiv preprint doi: https://doi.org/10.1101/2025.07.21.665862; this version posted July 22, 2025. The copyright holder for this preprint (which was not certified by peer review) is the author/funder, who has granted bioRxiv a license to display the preprint in perpetuity. It is made available under aCC-BY 4.0 International license.

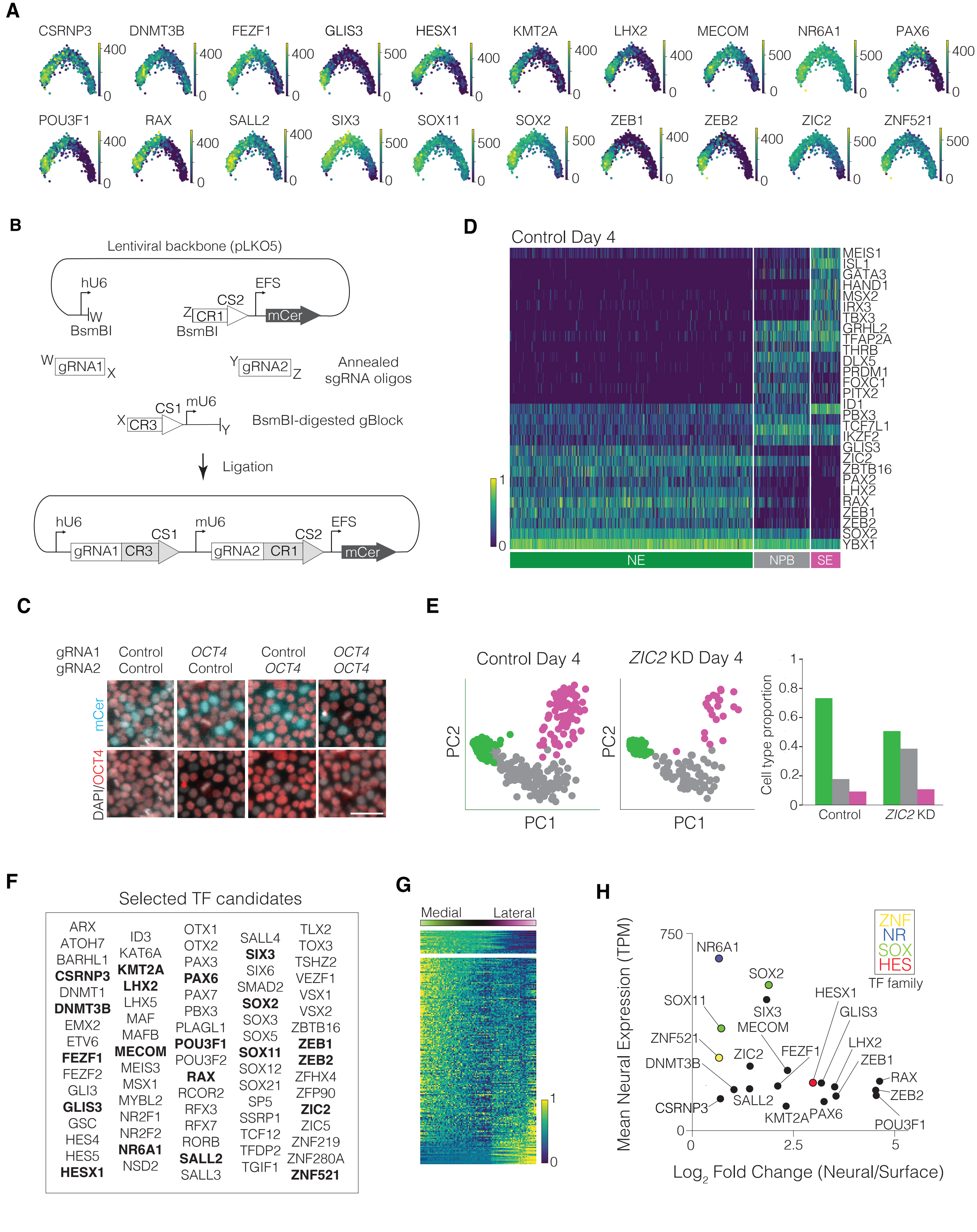

Figure S2

bioRxiv preprint doi: https://doi.org/10.1101/2025.07.21.665862; this version posted July 22, 2025. The copyright holder for this preprint (which was not certified by peer review) is the author/funder, who has granted bioRxiv a license to display the preprint in perpetuity. It is made available under aCC-BY 4.0 International license.

Figure S2. Transcriptome-based identification of candidate transcriptional regulators of anterior neurulation.

A) Principal component analysis of single-cell RNA-sequencing data from Day 2 organoids, colored by expression of 20 most medially expressed transcription factors. Gene expression is transcript count-normalized, and units are in transcripts per million. Scale bar for each sub-panel is shown to the right of the PCA plot.

B) Cloning schematic for lentiviral dual-guide RNA vector (based on Replogle, *et al*., 2020), for which pLKO5 backbone was modified to carry an mCerulean (mCer, a cyan fluorescent protein) reporter, digested with BsmBI, and ligated with two pairs of annealed sgRNA oligos together with a BsmBI-digested gBlock. The final vector encodes gRNA1-CR3-CS1 under a human U6 (hU6) promoter, gRNA2-CR1-CS2 under a mouse U6 (mU6) promoter, and mCerulean under an EFS promoter (Methods).

C) dCas9-KRAB CRISPRi H1 (WA01) transduced with dual-guide RNAs consisting of either a control non-targeting scrambled sequence ("control") or *OCT4*-targeting sequence ("*OCT4*") under the hU6 or mU6 promoter in guide RNA positions 1 ("gRNA1") and 2 ("gRNA2"), respectively, fixed and stained for DAPI and OCT4 48 hours post-transduction. Columns correspond to a control sequence at both positions (first column), an OCT4 sequence in position 1 and control sequence in position 2 (second column), a control sequence in position 1 and OCT4 sequence in position 2 (third column), and an OCT4 sequence at both positions (fourth column). Top and bottom rows correspond to merged channel images of the same fields of cells stained for OCT4 (red) and DAPI (gray), with or without the mCerulean channel (cyan) overlaid. The cells transduced with virus (mCer+) in the second, third, and fourth columns, but not those the first column, have low OCT4 levels, demonstrating that the *OCT4*-targeting guide is specific and functional when present in the first, second, or both positions. Scale bar: 50 µm.

D) scRNA-seq of Day 4 organoids transduced with a control non-targeting scramble dual-guide RNA construct. Control cells were clustered as neural ectoderm (NE, green), neural plate border (NPB, gray), or surface ectoderm (SE, magenta) based on expression of labeled transcription factors. Gene expression (color bar, bottom left) is normalized to median transcript count, followed by log- and min-max normalization.

E) Left: Cells from scRNA-seq of Day 4 scramble control and *ZIC2* knockdown organoids plotted along the first two principal components for control cells. The data points corresponding to the three cell types are colored appropriately (NE, green; NPB, gray; SE, magenta). Right: Histogram of cell type proportions shows that *ZIC2* knockdown organoids have less NE, more NPB, and a similar SE fraction compared to control organoids.

F) Full list of 78 transcription factor candidates selected for knockdown, with 20 neural transcription factors selected based on mediolateral expression (Figure 2A, S2A) in bold.

G) Standardized mediolateral expression profiles of top 200 most highly expressed transcription factors in Day 2 organoids, ordered from most medially expressed (top) to most laterally

bioRxiv preprint doi: https://doi.org/10.1101/2025.07.21.665862; this version posted July 22, 2025. The copyright holder for this preprint (which was not certified by peer review) is the author/funder, who has granted bioRxiv a license to display the preprint in perpetuity. It is made available under aCC-BY 4.0 International license.

expressed (bottom). The 20 most medially expressed transcription factors (Figure 2A, right) are separated above the rest.

H) Plot of mean expression in neural ectoderm (transcripts per million, TPM) versus log fold change in expression between the neural and surface ectoderm in Day 2 organoids for the top 20 mediolaterally enriched transcription factors from Figure 2E. For all 20 transcription factors, mean expression > 70 TPM and log2(fold change) > 0.6.

bioRxiv preprint doi: https://doi.org/10.1101/2025.07.21.665862; this version posted July 22, 2025. The copyright holder for this preprint (which was not certified by peer review) is the author/funder, who has granted bioRxiv a license to display the preprint in perpetuity. It is made available under aCC-BY 4.0 International license.

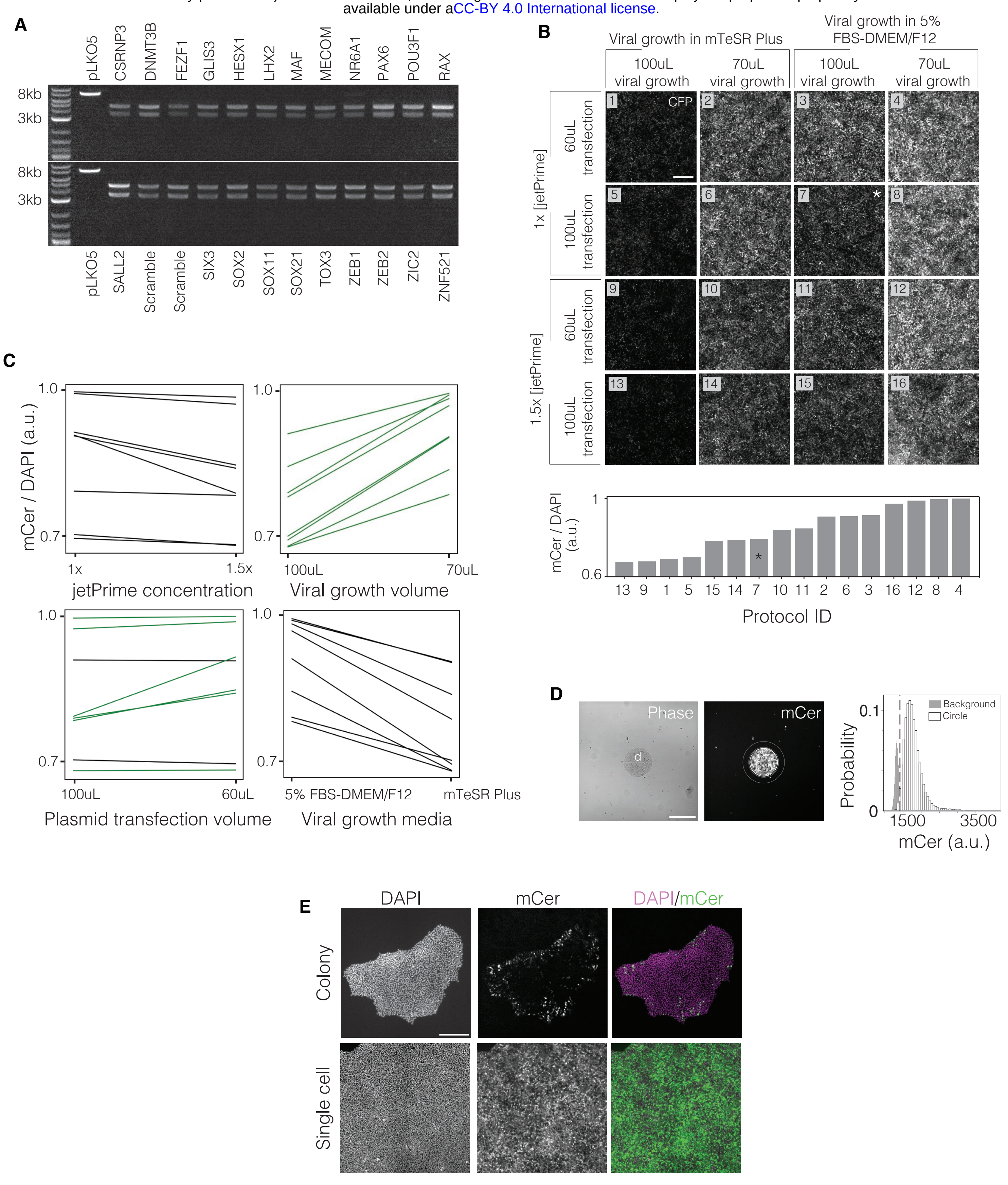

Figure S3

bioRxiv preprint doi: https://doi.org/10.1101/2025.07.21.665862; this version posted July 22, 2025. The copyright holder for this preprint (which was not certified by peer review) is the author/funder, who has granted bioRxiv a license to display the preprint in perpetuity. It is made available under aCC-BY 4.0 International license.

Figure S3. Development of transfection and transduction methods.

(A) Gel electrophoresis of non-clonal plasmid stocks (see Figure S2B, Methods) after double digest with BamHI and DraIII. Expected band length for backbone (control lane): 7.4kb. Expected band lengths for backbone with ligated inserts (following lanes): 3.4kb and 4.5kb. Ladder: NEB 1kB Plus. Band intensities show that a majority of non-clonal plasmid stock contains the desired insert.

(B) Top: Epifluoresence images of mCerulean (mCer) expression in confluent hESC culture, 48 hours after lentiviral transduction with scramble control vector from one of 16 different HEK transfection protocols. Protocols altered 4 variables in a 96-well transfection of HEK cells. Bottom: Ratio of mCer to DAPI signal from each protocol above, rank ordered by transduction efficiency. Protocol 7 (*) is the standard protocol recommended by jetPrime. Scale bar: 300 µm.

(C) Ratio of mCer to DAPI signal from Figure S3B, paired by shared variables to show the effect of changing the variable in each controlled pair. Changes that resulted in increased transduction are shown in green. General trends show that decreasing viral growth volume and decreasing plasmid transfection volume result in higher viral production.

(D) Example of transduction efficiency analysis. Left: Colony diameter as measured by colony segmentation by Ilastik in the phase channel. Middle: Colony segmentation as applied to the mCer channel (white) and 2D background halo (area between gray circles). Right: Quantification of mCer signal from colony (white) and background halo (gray). Dotted line (threshold) represents 95th percentiles of mCer expression in the background halo. This approach is validated with nuclear analysis in Figure 3C. Scale bar: 300 µm.

(E) mCer expression in hESC colonies, 48 hours after transduction with scramble control vector made with Protocol 4 from Figure S3B. Top: hESCs were transduced after 48 hours of adherent growth on plates. Bottom: hESCs were transduced in single cell suspension directly after dissociation. Scale bar: 300 µm.

bioRxiv preprint doi: https://doi.org/10.1101/2025.07.21.665862; this version posted July 22, 2025. The copyright holder for this preprint (which was not certified by peer review) is the author/funder, who has granted bioRxiv a license to display the preprint in perpetuity. It is made available under aCC-BY 4.0 International license.

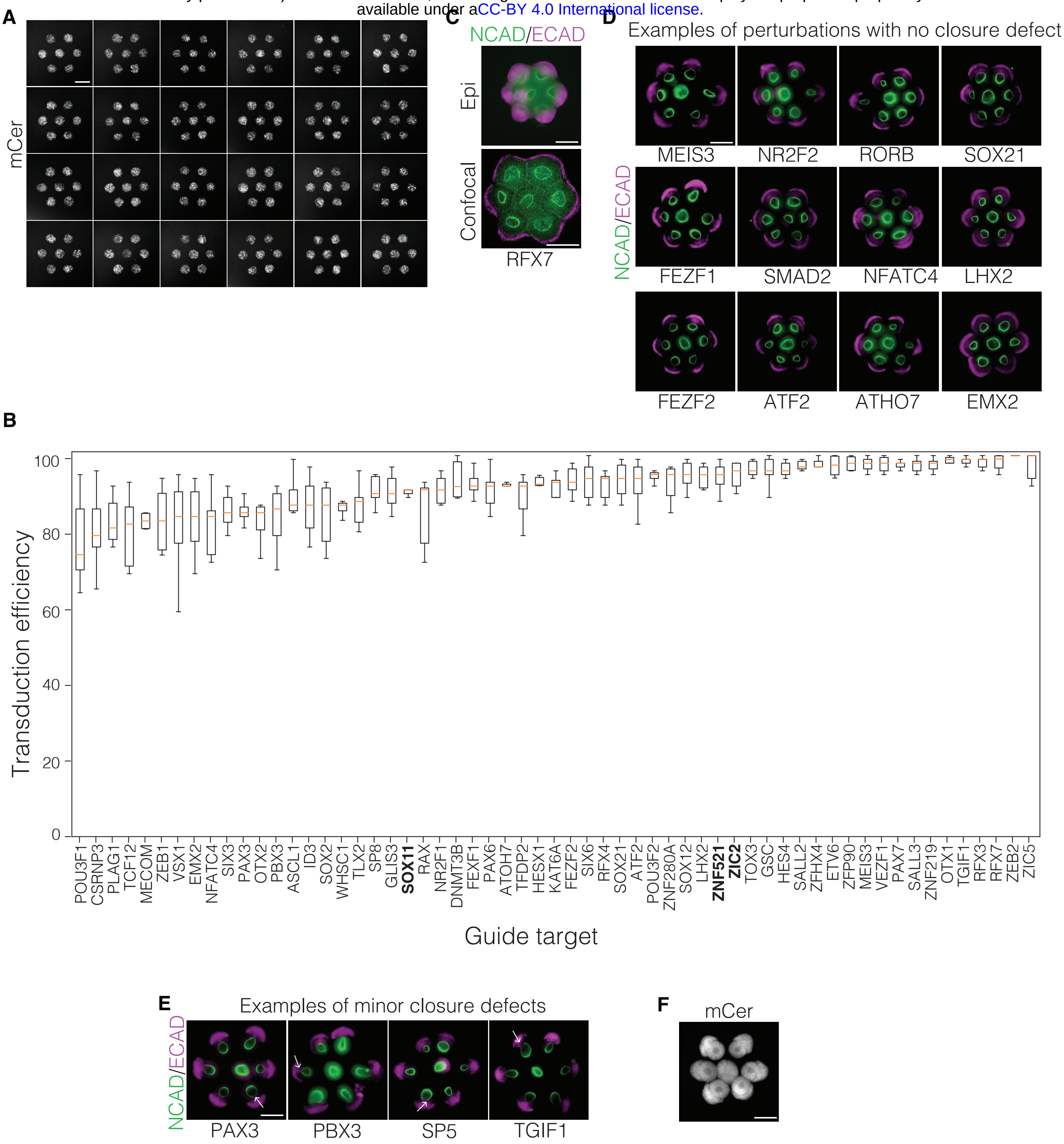

Figure S4

bioRxiv preprint doi: https://doi.org/10.1101/2025.07.21.665862; this version posted July 22, 2025. The copyright holder for this preprint (which was not certified by peer review) is the author/funder, who has granted bioRxiv a license to display the preprint in perpetuity. It is made available under aCC-BY 4.0 International license.

Figure S4. Perturbation of most transcription factors does not give rise to morphological phenotypes.

(A) 24 examples of live epifluorescence microscopy of lentiviral marker mCerulean (mCer), 48 hours after transduction in suspension and seeding on micropatterns as in Figure 3D. Scale bar: 300 µm.

(B) Quantification of transduction efficiencies from individual sgRNAs, 48 hours after transduction in suspension and seeding on micropatterns as in Figure 3D. The median transduction efficiency across all guides using the non-purified plasmid prep was 93%.

(C) Top: Epifluorescence image of *RFX7* perturbation. Bottom: Maximum intensity projection of a confocal Z-stack (bottom). All organoids received a score of 0 (no phenotype) after confocal imaging. Scale bar: 300 µm.

(D, E) Epifluorescence microscopy of terminal organoid morphologies, differentiated as in Figure S1D for 6 days, then fixed and immunostained. E: Examples of minor openings are indicated in white arrows. Scale bar: 300 µm.

(F) Distribution of mCer in organoids transduced with a scramble guide, measured 8 days post-transduction (2 days of mTeSR growth and 6 days of differentiation). Scale bar: 300 µm.

bioRxiv preprint doi: https://doi.org/10.1101/2025.07.21.665862; this version posted July 22, 2025. The copyright holder for this preprint (which was not certified by peer review) is the author/funder, who has granted bioRxiv a license to display the preprint in perpetuity. It is made available under aCC-BY 4.0 International license.

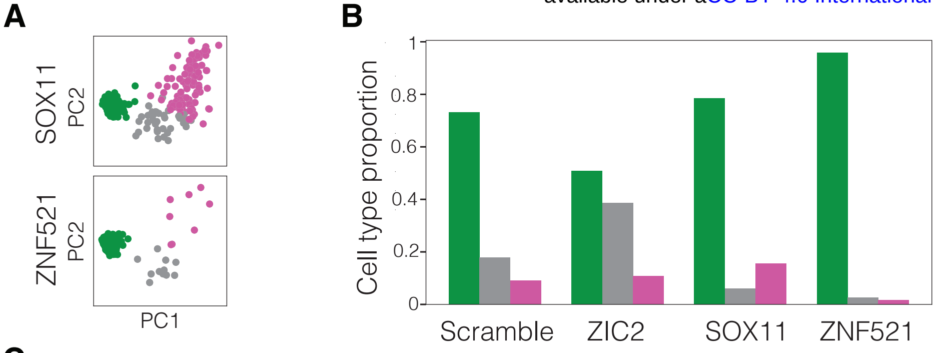

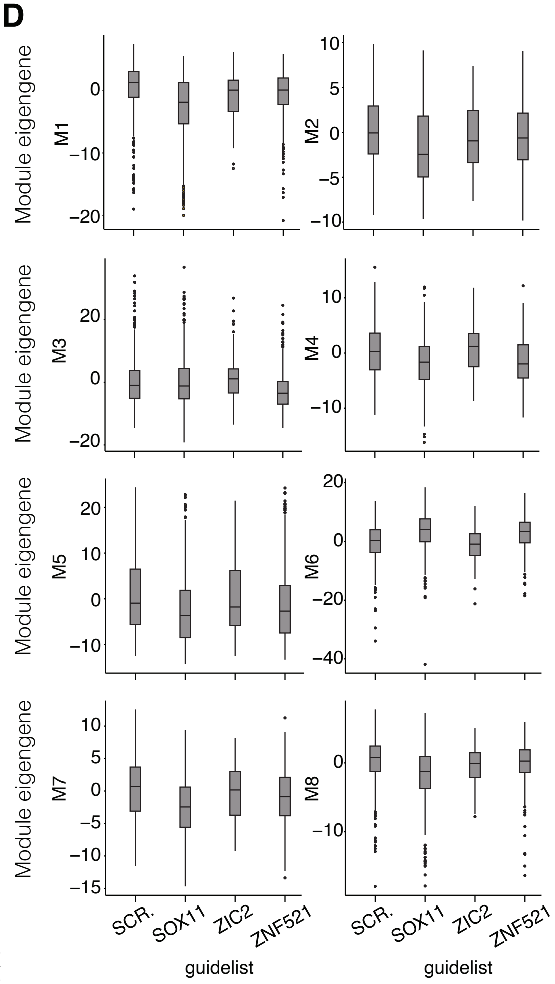

C

Non-phenotype conditions

Guide target	Number of cells collected	Mean expression in Scramble cells (tpm)	Fold change in guide-expressing cells	Significant by Anderson-Darling test (p<0.05)
OTX2	20	77	0.48	True
PAX6	20	88	0.57	True
POU3F1	21	90	0.05	True
LHX2	26	167	0.50	True
TGIF1	19	59	0.08	True
TOX3	37	132	0.43	True
GLI3	44	244	0.44	True
ZFHX4	30	163	1.08	False

Phenotype conditions

Guide target	Number of cells collected	Mean expression in Scramble cells (tpm)	Fold change in guide-expressing cells	Significant by Anderson-Darling test (p<0.05)
ZIC2	104	198	0.40	True
SOX11	489	378	0.07	True
ZNF521	473	322	0.58	True

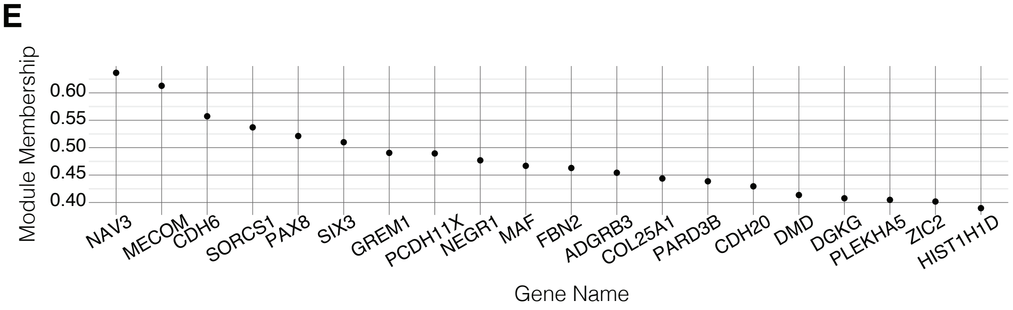

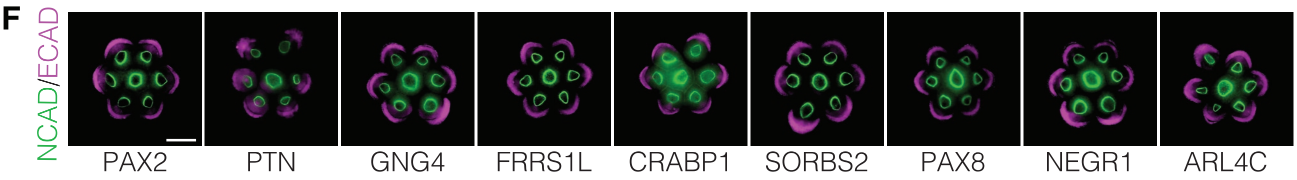

Figure S5

bioRxiv preprint doi: https://doi.org/10.1101/2025.07.21.665862; this version posted July 22, 2025. The copyright holder for this preprint (which was not certified by peer review) is the author/funder, who has granted bioRxiv a license to display the preprint in perpetuity. It is made available under aCC-BY 4.0 International license.

Figure S5. Analysis of scRNA-seq data from perturbed organoids.

(A) Principal component analysis of cell types in Day 4 organoids infected guides against *SOX11* or *ZNF521*. Cells are plotted using the two major principal components from the scramble control data from Figure S2E. Cell type assignments to neural ectoderm (green), neural plate border (gray), and surface ectoderm (magenta) are based on expression of canonical transcription factors from scramble control data in Figure S2D.

(B) Quantification of cell type proportions from scRNA-seq of all control and major closure defect datasets (*ZIC2*, *SOX11*, and *ZNF521* knockdown). Cell types are assigned as in (A).

(C) Top: Knockdown efficacy calculations of sgRNAs that led to no phenotype or minor closure defects. Seven out of eight guides showed significant knockdown of their target gene with a knockdown efficacy of >40%. Bottom: Knockdown efficacy calculation of sgRNAs that produced a major closure defect. All three guides showed significant knockdown of their target gene with a knockdown efficacy of >40%.

(D) Dendrogram module eigengene values from the remaining modules from Figure 5C. No other module shows significant changes in expression in any perturbation condition tested.

(E) Correlation values with module eigengene (module membership) of top 20 genes in Module 9, including ZIC2, from Figure 5C.

(F) Epifluorescence images of Day 4 organoids perturbed with guides against downstream targets of *SOX11*, *ZIC2*, and/or *ZNF521*. Scale bar: 300 µm
